# Green and Efficient Synthetic Protocol for 1,3,5-Triazine Derivatives with Anticancer Potential Against Colorectal Cancer

**DOI:** 10.3390/molecules30112437

**Published:** 2025-06-02

**Authors:** Julia Chrzan, Anna Karolina Drabczyk, Izabela Siemińska, Monika Baj-Krzyworzeka, Katarzyna Ewa Greber, Jolanta Jaśkowska, Damian Kułaga, Krzesimir Ciura

**Affiliations:** 1 Department of Organic Chemistry and Technology, Faculty of Chemical Engineering and Technology, Cracow University of Technology, 24 Warszawska Street, 31-155 Cracow, Poland; juliachrzan9@o2.pl (J.C.); anna.drabczyk@pk.edu.pl (A.K.D.); izabela.sieminska@urk.edu.pl (I.S.); jolanta.jaskowska@pk.edu.pl (J.J.); 2 Faculty of Veterinary Medicine, University of Agriculture in Cracow, 24/28 Mickiewicza Street, 30-059 Cracow, Poland; 3 Department of Clinical Immunology, Medical College, Jagiellonian University, 30-663 Krakow, Poland; monika.baj-krzyworzeka@uj.edu.pl; 4 Department of Physical Chemistry, Faculty of Pharmacy, Medical University of Gdansk, 107 General Jozef Haller Avenue, 80-416 Gdansk, Poland; katarzyna.greber@gumed.edu.pl; 5 Laboratory of Environmental Chemoinformatics, Faculty of Chemistry, University of Gdansk, 63 Wita Stwosza Street, 80-308 Gdansk, Poland

**Keywords:** 1,3,5-triazine, green chemistry, microwave-assisted synthesis, sonochemistry, colorectal cancer, anticancer agents, phase-transfer catalysis, cytotoxicity, ADME, drug likeness

## Abstract

Colorectal cancer (CRC) remains a major global health challenge, necessitating the development of more effective and environmentally sustainable treatments. This study presents a novel green synthetic protocol for 1,3,5-triazine derivatives with anticancer potential, employing both microwave-assisted and ultrasound-assisted methods. The synthesis was optimized using 4-chloro-*N*-(2-chlorophenyl)-6-(morpholin-4-yl)-1,3,5-triazin-2-amine as the key intermediate, with sodium carbonate, TBAB, and DMF providing optimal yields under microwave conditions. To enhance sustainability, a modified sonochemical method was also developed, enabling efficient synthesis in aqueous media with a minimal use of organic solvents. A series of nine morpholine-functionalized derivatives were synthesized and evaluated for cytotoxic activity against SW480 and SW620 colorectal cancer cell lines. Compound **11** demonstrated superior antiproliferative activity (IC₅₀ = 5.85 µM) compared to the reference drug 5-fluorouracil, while compound **5** showed promising dual-line activity. In silico ADME analysis supported the drug likeness of the synthesized compounds, and biomimetic chromatography analysis confirmed favorable physicochemical properties, including lipophilicity and membrane affinity. These results underscore the potential of the developed protocol to produce bioactive triazine derivatives through an efficient, scalable, and environmentally friendly process, offering a valuable strategy for future anticancer drug development.

## 1. Introduction

Colorectal cancer (CRC) is among the most prevalent malignancies worldwide, ranking third in incidence and second in cancer-related mortality. Annually, approximately 1.93 million new cases are diagnosed, with over 900,000 deaths attributed to the disease [1]. Due to its substantial impact, CRC poses a significant burden on both healthcare systems and affected individuals. One of the primary challenges in managing CRC is the asymptomatic nature of early-stage disease, which frequently leads to delayed diagnosis. Consequently, early detection is critical for reducing mortality rates. Although colonoscopy remains the gold standard for CRC screening, there is increasing interest in non-invasive diagnostic approaches, such as liquid biopsy, which may facilitate the earlier identification of malignant transformations [2].

Despite the availability of conventional treatment modalities, including surgery, radiotherapy, and chemotherapy, CRC management remains challenging. These therapeutic strategies often yield suboptimal outcomes, with issues such as tumor recurrence and resistance to treatment posing significant hurdles. Currently used chemotherapeutic agents, such as fluoropyrimidine (5-fluorouracil, 5-FU), oxaliplatin, and irinotecan, exhibit limited efficacy and are frequently associated with severe adverse effects. This underscores the urgent need for the development of more targeted and effective treatment strategies [3,4,5].

In recent years, novel therapeutic approaches, including immunotherapy, targeted therapies, and nanomedicine, have demonstrated potential in improving CRC treatment outcomes. Some of these strategies have already been integrated into clinical practice, while others continue to show promise in clinical trials, offering new perspectives for enhancing CRC management and patient prognosis [6,7,8]. 1,3,5-triazine and its derivatives are key compounds in the development of chemotherapeutic drugs, exhibiting significant biological activity, including antibacterial [9], antifungal [10], antimalarial [11], anticancer [12], antiviral [13], antimicrobial [14], anti-inflammatory [15], and antitubercular [16] properties. Its derivatives also find applications as dyes [17], lubricants [18], and analytical reagents [19]. 1,3,5-triazines represent a promising class of chemical compounds with confirmed anticancer activity against colorectal cancer. Numerous studies have shown that their mechanism of action may involve both the inhibition of key enzymes and the induction of apoptosis in cancer cells.

**Figure 1:**
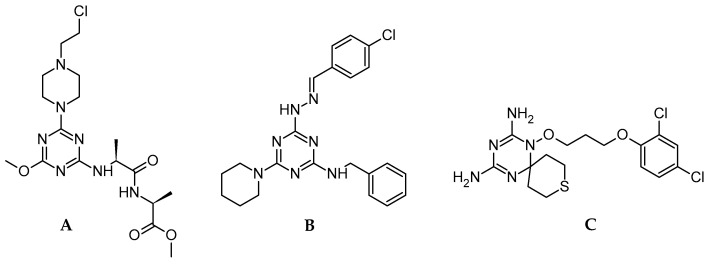
Examples of 1,3,5-triazine derivatives (**A**–**C**) with confirmed biological activity against colon cancer.

Studies on 1,3,5-trisubstituted derivatives (Figure 1) have demonstrated that some of these compounds exhibit high cytotoxicity against colorectal cancer cell lines such as DLD-1, HCT-116, and HT-29. An example is methyl(4-(4-(2-chloroethyl)piperazin-1-yl)-6-methoxy-1,3,5-triazin-2-yl)-L-alanyl-L-alaninate (Figure 1A), which shows strong anticancer activity. The mechanism of action of this compound includes the inhibition of cancer cell proliferation and the induction of apoptosis through the attenuation of key signaling pathways [12]. A new series of compounds (Figure 1B) based on the s-triazine core and Schiff base moieties exhibits strong antiproliferative activity against HCT-116 cells. Studies have shown that the most active derivatives were those with piperidine and benzyl substituents, achieving IC₅₀ values in the range of 3.64–5.60 µM. Additionally, it has been demonstrated that the introduction of electrophilic substituents, such as chlorine or bromine, enhances the cytotoxicity of these compounds [20]. Some triazines could inhibit enzymes involved in tumorigenesis. An example is a dihydrofolate reductase (DHFR) inhibitor (Figure 1C), which suppresses the proliferation of HCT-116 cells. Moreover, triazines may modulate the activity of enzymes associated with protein ubiquitination and cell cycle regulation [21].

Cyanuric chloride is the most used precursor for synthesizing a variety of compounds featuring the 1,3,5-triazine core. The synthesis of substituted triazine derivatives (mono-, di-, and tri-substituted) via the nucleophilic substitution of cyanuric chloride requires careful control of reaction temperature. The substitution of the first chlorine atom typically occurs at low temperatures, around 0 °C, while the second chlorine atom is replaced at room temperature. This sequential substitution is facilitated by the strong electron-accepting nature of chlorine atoms in cyanuric chloride, which enhances nucleophilic attack. The third substitution occurs at elevated temperatures, typically in the range of 70–100 °C. This stepwise reactivity allows for the introduction of three distinct nucleophiles within the 1,3,5-triazine core, enabling the generation of a diverse array of promising derivatives with broad applications [22]. This stage is typically carried out using conventional heating methods. However, this approach demands a significant amount of energy, extends reaction times, and consequently increases overall costs. Additionally, the excessive consumption of electricity and cooling water makes this technique environmentally unsustainable. While these challenges may be less apparent at a laboratory scale, they become critical when applied to industrial processes, where production costs escalate considerably. Despite its simplicity, conventional heating is not the most efficient strategy and introduces further complications, particularly regarding solvent usage. Many of the solvents commonly employed in these reactions are not only costly but also pose environmental and safety concerns, requiring proper handling and disposal. Given these limitations, traditional synthetic routes for obtaining 1,3,5-triazine derivatives fail to meet the principles of sustainable and green chemistry, emphasizing the need for more innovative and eco-friendly alternatives [23,24,25]. Notably, the final stage of substitution can be effectively conducted not only at the boiling point of the solvent but also under more alternative and green approaches like microwave irradiation or sonochemistry protocol, further expanding the synthetic possibilities.

Díaz-Ortiz et al. compared the microwave-assisted method with the conventional approach, demonstrating that microwave irradiation reduced the reaction time from 5 days to 10 min. The reaction was carried out without a solvent, in the presence of excess amines neutralizing HCl, achieving a yield of up to 62%. In contrast, the conventional method required the use of THF and DIPEA. Additionally, the effect of DMSO as a solvent on the reaction process was analyzed [26]. Lim et al. developed a microwave-assisted method in ethanol, utilizing HCl as a catalyst and NaOH in a subsequent reaction step. The optimization of reaction conditions revealed that a temperature of 180 °C and a reaction time of 20 min led to high yields [27]. Shahari et al. conducted a microwave-assisted reaction in ethanol, using HCl as a catalyst and NaOH in a later step. The process was carried out at 140 °C, allowing for high product purity and yields of up to 71% [28]. Kułaga et al. developed a microwave-assisted method using DMF as the solvent and TBAB as a phase-transfer catalyst (PTC). The reaction lasted only 150 s, achieving yields of up to 88%, with PTC significantly improving process efficiency [29].

Kułaga et al. also developed a sonochemical method for the synthesis of 1,3,5-triazine derivatives, demonstrating that ultrasonic irradiation significantly shortens the reaction time to 5 min while maintaining high yields (>75%). The reactions were carried out in water, using sodium carbonate as a base and TBAB as a phase-transfer catalyst, with ultrasound applied via a probe sonicator at 70% amplitude. This approach provided high-purity products and was shown to be significantly greener than the classical method [29]. Verma et al. synthesized N^2^,N^4^,N^6^-tris((pyridin-2-ylamino)methyl)-1,3,5-triazine-2,4,6-triamine via ultrasonication in ethanol using melamine, formaldehyde, and 2-aminopyridine in a 1:3:3 molar ratio. The reaction under ultrasonic conditions at room temperature proceeded within 30–35 min and achieved 84% yield, outperforming the conventional method, which required 5–6 h of reflux and gave 69% yield [30]. Al-Rasheed et al. applied ultrasonic irradiation at 40 °C for 30–60 min to synthesize 4,6-disubstituted-1,3,5-triazine hydrazone derivatives in ethanol with catalytic acetic acid. Compared to the conventional reflux method (4–5 h), the sonochemical protocol significantly improved the reaction rate and product yield (up to 96%) [31].

The combination of phase transfer catalysis and green approaches offers a modern way to optimize chemical synthesis processes by combining efficient reagent transfer with accelerated reactions. PT catalysts, using ammonium or phosphonium salts, facilitate the transfer of reagents between the organic and aqueous phases, promoting faster and more selective reactions. In 1,3,5-triazine synthesis, cyanuric chloride activation by TBAB accelerates nucleophilic substitution, leading to the more efficient formation of desired compounds. Additionally, the use of microwaves or sonochemistry significantly shortens reaction times, reducing them from several hours to just minutes, which translates into lower energy consumption and reduced waste. This methodology also allows for the rapid screening of various reaction conditions, minimizing the use of reagents and solvents. Combined with PTC, green approaches enable reactions to be carried out under milder conditions, increasing yields and product selectivity. The protocol makes the synthesis processes not only more efficient but also more sustainable, which is crucial in industrial chemical applications, where optimizing efficiency and reducing waste are key considerations [32,33,34].

**Figure 2:**
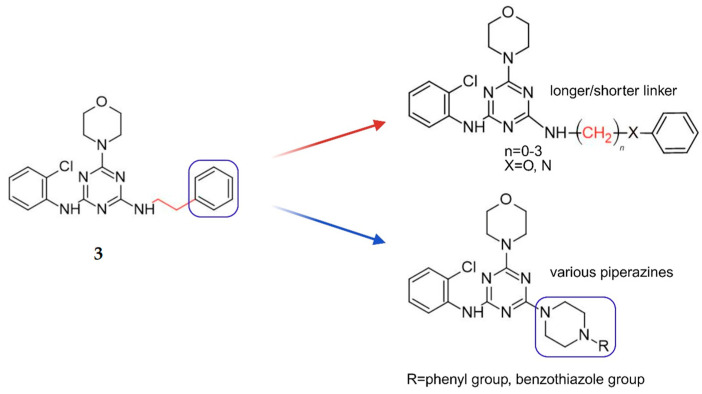
Designing new compound.

Based on the above considerations, it is justified to undertake research aimed at developing a universal, eco-friendly, and cost-effective method for synthesizing 1,3,5-triazine derivatives, specifically using microwave-assisted synthesis or sonochemistry. This approach offers several advantages: it ensures higher reaction yields, shorter reaction times, and aligns with the principles of green chemistry by reducing energy consumption and the need for harmful solvents. Additionally, the green approach can improve selectivity and accelerate reactions, making it a promising strategy for the large-scale and sustainable production of these compounds. This work continues our previous research on green and sustainable synthetic methodologies, in which we compared the efficiency of two leading approaches: microwave-assisted [33] and ultrasound-assisted synthesis [29]. Among these, ultrasonic irradiation demonstrated clear advantages in terms of reaction efficiency and environmental impact. In this study, we initially optimized the conditions for a model reaction under microwave irradiation using 2-phenylethylamine (**2**) as a reagent and then validated the method using ultrasound synthesis. This approach was guided by the well-documented therapeutic potential of 1,3,5-triazine derivatives in the treatment of cancer, particularly colorectal cancer [20,21]. Based on the developed synthetic methodology, a small library of compounds was obtained (Figure 2). To demonstrate the versatility of the process, the library was also re-synthesized using an ultrasound-assisted method, confirming the feasibility of an alternative, more rapid, and environmentally friendly synthetic route. The literature data suggest that hybrid molecules combining the 1,3,5-triazine core with phenylethylamine moieties may exert antiproliferative effects against cancer cells [35,36]. The chosen model reaction thus serves as a representative system to explore the synthesis of 1,3,5-triazine derivatives that could be further developed as anticancer agents.

Given the promising biological activity of 1,3,5-triazine derivatives reported in the literature, we sought to further investigate their potential by evaluating the newly synthesized compounds in biological assays. To this end, the obtained derivatives were evaluated for their antiproliferative activity against two colorectal cancer cell lines: SW480 and SW620. To assess their drug-like properties, we conducted in silico ADMET studies, providing a preliminary evaluation of the pharmacological potential of the synthesized compounds. In addition, key physicochemical parameters, such as lipophilicity and phospholipid affinity, were determined experimentally.

## 2. Results and Discussion

### 2.1. Chemistry

**Scheme 1:**
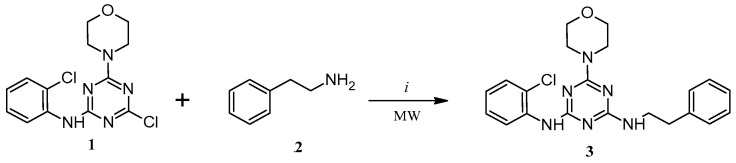
Microwave synthesis of compound **3**. MW—microwave synthesis. *i*—base:/K_2_CO_3_/KOH/NaOH/DIPEA/TEA/NH_3_∙H_2_O; PTC: TBAB/TEBA/TEAC/CTBA/KI; solvent: DMF/H_2_O.

The molecules that were selected for synthesis were morpholine-functionalized 1,3,5-triazine derivatives as compounds with potential anticancer activity [37,38]. A series of test reactions between 4-chloro-*N*-(2-chlorophenyl)-6-(morpholin-4-yl)-1,3,5-triazin-2-amine (**1**) and 2-phenylethylamine (**2**) were performed using a 50 W microwave reactor at 150 °C for 2.5 min according to Scheme 1. Various bases (Table 1, Reactions No 1–7) were tested including seven different bases: sodium carbonate—Na_2_CO_3_, potassium carbonate—K_2_CO_3_, potassium hydroxide—KOH, sodium hydroxide—NaOH, *N*,*N*-diisopropylethylamine—DIPEA, triethylamine—TEA, ammonia aq.—NH_3_∙H_2_O. In the next step (Table 1, Reactions No 8–11), four different phase transfer catalysts were tested including tetrabutylammonium bromide—TBAB, benzyltriethylammonium chloride—TEBA, tetraethylammonium chloride—TEAC, cetyltrimethylammonium bromide—CTBA, potassium iodide—KI, and one reaction without PTC (Table 1, Reaction No 12). In the final stage of the optimization process, the influence of two different solvents (*N,N*-dimethylformamide—DMF—Table 1, Reaction No 1; water—H_2_O—Table 1, Reaction No 13) was evaluated as well as Reaction 14 without any solvent (Table 1). Based on the most favorable selected reaction conditions, further experiments were performed.

The tested bases (Table 1) can be classified in the following pattern: (a) strong inorganic bases (NaOH, KOH); (b) weak inorganic bases (Na_2_CO_3_, K_2_CO_3_, and NH_3_∙H_2_O), and (c) organic bases (DIPEA, TEA). Na₂CO₃ was the most effective inorganic base, yielding 87%, whereas NH₃*H₂O was the least favorable (2% yield). In this case, the reaction 7 yield was 2%. It may be explained by the fact that, during the reaction process, ammonia is vaporized, and, to increase yield, the reaction should be carried out in a closed-seal tube reactor. Of the two organic bases with which reactions were carried out, the more favorable one was TEA, with a yield of 56%, compared to DIPEA, where the yield reached 23%. Of all the bases tested, the weaker inorganic bases, primarily Na_2_CO_3_, were chosen for further study due to their low environmental toxicity and ease of disposal, in extraction with water.

Reaction efficiency varied depending on the phase transfer catalyst used (Table 1). The lowest yield was observed when potassium iodide (KI) was used as an inorganic catalyst, resulting in an efficiency of 44%. Among the organic catalysts, TEBA proved to be the least effective, yielding 56%. A reaction without a phase transfer catalyst was also conducted, achieving an efficiency of 53%, which was higher than that of KI and TEBA. In contrast, TEAC significantly improved the reaction efficiency, reaching 80%, while the highest yield within Table 1—85%—was obtained using CTBA in combination with Na₂CO₃ as a base and DMF as a solvent under microwave conditions. However, comparing the influence of PTC used in reaction 1 and reactions 8–11, the best result was achieved with TBAB (Table 1), which provided the highest efficiency of 87%. Due to its superior performance compared to all other tested catalysts, TBAB was selected for further studies.

The influence of different solvents on the reaction efficiency was evaluated (Reactions No. 13 and 14). The lowest yield was observed when the reaction was conducted without a solvent, resulting in 8% efficiency (Reaction No. 14). A slightly higher yield of 10% was obtained when water was used as the solvent in reaction 13. The highest efficiency was achieved using DMF (Table 1, Reaction No. 1), with a yield of 87%.

**Scheme 2:**
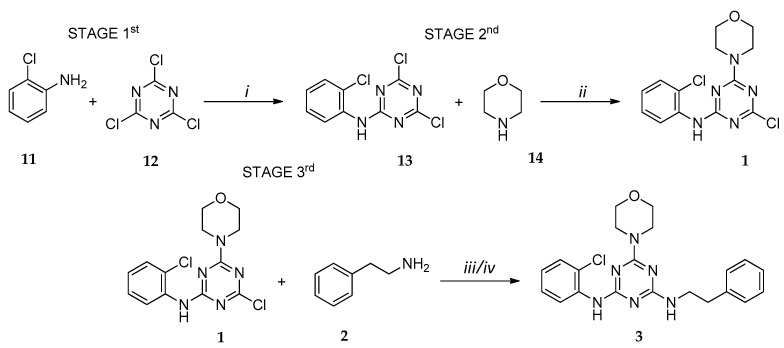
Stages of synthesis of derivatives of 1,3,5-triazine. *i*—THF, DIPEA, 0 °C; *ii*—THF, DIPEA, and RT; *iii*—Na_2_CO_3_, TBAB, DMF, and MW: 2,5 min, 50 W; *iv*—Na_2_CO_3_, TBAB, H_2_O and ))), room temp., A = 70% MW—microwave synthesis; )))—sonochemistry.

Having optimized the reaction conditions, we proceeded to evaluate the applicability of the developed method in the synthesis of a series of derivatives (Table 2). The target compounds were obtained through a three-step reaction sequence (Scheme 2). In the first step, the alkylation of 2-chloroaniline (**11**) with cyanuric chloride (**12**) was carried out in tetrahydrofuran (THF) at a temperature below 0 °C, affording intermediate **13** with a yield of 49%. The second step involved the reaction of 4,6-dichloro-*N*-(2-chlorophenyl)-1,3,5-triazin-2-amine (**13**) with morpholine (**14**) in the presence of *N*,*N*-diisopropylethylamine (DIPEA) as a base and THF as the solvent obtaining compound **1**. In the final step, intermediate **1** was subjected to nucleophilic substitution with selected amines (**2a**–**h**) under optimized conditions. Based on the preliminary optimization studies, sodium carbonate (Na₂CO₃) was selected as the base, TBAB as the phase-transfer catalyst, and DMF as the solvent. These conditions were subsequently applied to the synthesis of the remaining derivatives (**4**–**11**) under microwave irradiation at 150 °C and 50 W for 150 s. The final compounds were obtained with yields ranging from 54% to 87%, demonstrating the efficiency and versatility of the developed methodology.

The above experiments demonstrate that the developed method for the synthesis of 1,3,5-triazines functionalized with morpholine is both universal and efficient. However, it should be noted that DMF is toxic, emphasizing the need for its elimination or the search for alternative, less harmful solvents. Therefore, the use of DMF as a solvent does not allow for a full classification of the protocol as green-friendly.

An alternative strategy to address the toxicity of DMF involves the application of a sonochemical approach, which is considered a green and sustainable technique. The literature reports provide evidence that certain reactions can be successfully performed in aqueous media under ultrasonic irradiation [39,40,41]. Based on these findings, we conducted a model reaction (Scheme 1) under sonochemical conditions using Na_₂_CO₃ as the base, TBAB as the phase transfer catalyst, and water as the solvent [29]. Under these conditions, compound **3** was obtained in a 73% yield, with 23% of unreacted starting material **1.** Due to the presence of unreacted substrate, we decided to use DMF in a catalytic amount not exceeding 5% by weight of the total reaction mixture. In this system, DMF may act as an energy transfer facilitator and improve the interaction between immiscible reaction phases, thereby enhancing the efficiency of the phase-transfer catalysis. The addition of a small amount of DMF led to an increase in the reaction yield from 73% to 90% of compound **3**.

Subsequently, based on a slightly modified version of the original procedure [29], we re-synthesized the previously obtained compounds and compared their yields (Table 2). It was found that the ultrasound-assisted method was equally effective as the microwave-assisted synthesis, and, in some cases, even superior. An exception was compound **10**, which was obtained with a yield of 41% under ultrasound conditions, while its synthesis via the microwave method resulted in a higher yield of 60%. Moreover, the use of water as the main solvent with only a catalytic amount of DMF, combined with an alternative method of energy input, makes the ultrasound-assisted approach an attractive and, above all, fully green alternative for conducting organic reactions.

### 2.2. In Vitro Results and ADME Evaluation

The in vitro screening of nine synthesized 1,3,5-triazine derivatives (**3–11**) was conducted against two colorectal cancer cell lines: SW480, SW620 with 5-fluorouracil serving as a reference compound (Table 3). The results revealed notable structure–activity relationships, shedding light on the influence of structural modifications on cytotoxic activity.

Among the tested compounds, **11** exhibited the most potent antiproliferative effect against the SW620 cell line, with an IC_50_ value of 5.85 µM, surpassing the activity of 5-fluorouracil (21.74 µM). Similarly, **5** demonstrated promising and stable cytotoxicity towards both cell lines SW480 (IC_50_ = 43.12 µM) and SW620 (IC_50_ = 32.83 µM), indicating that the benzyloamine substitution contributes positively to the biological activity.

In contrast, **3**, **6, 7** and **10** exhibited the highest IC_50_ values across all cell lines, indicating limited cytotoxic potential. Additionally, **9** was found to be inactive against the SW480 cell line but with some activity against SW620. Compounds **5**, **8,** and **11** emerge as promising candidates for further investigation, demonstrating superior activity against the aggressive SW620 or SW480 cell line and warranting additional studies to elucidate their mechanisms of action.

**Figure 3:**
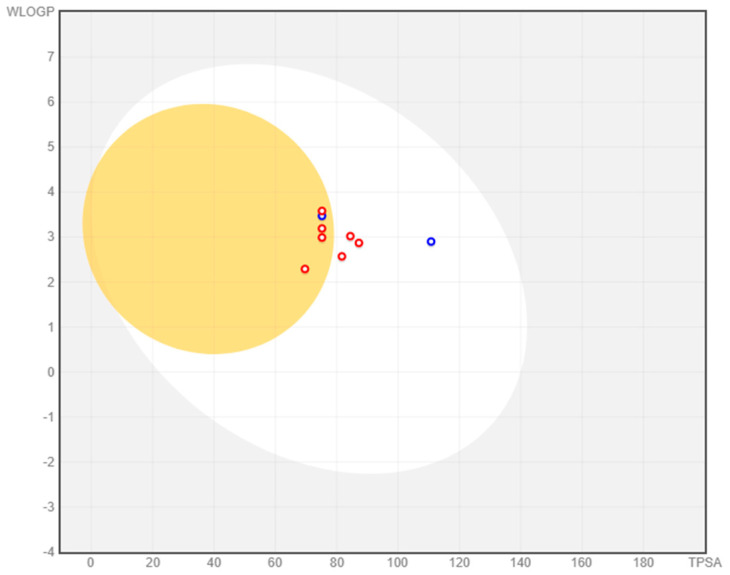
The predictive “BOILED-Egg” model for the compounds under investigation. The yellow region illustrates the probability of crossing the blood–brain barrier (BBB), whereas the white region indicates predicted gastrointestinal absorption. Blue dots represent P-gp effluxed compounds from the CNS. Red dots represent compounds not to be effluxed by P-gp from the CNS.

Initial ADME assessments were conducted alongside the design of the synthesized structures, utilizing in silico tools such as ADMET-AI, SwissADME, and Percepta [42]. While nearly all compounds met standard drug-likeness criteria, including adherence to Lipinski’s *Rule of Five* and acceptable molecular weights, lipophilicity, and hydrogen-bonding patterns, only a few exhibited molecular weights exceeding 500. However, for anticancer agents, alternative criteria may be considered, as standard drug-likeness guidelines primarily apply to molecules intended for oral administration, whereas anticancer agents can be administered in other ways. Nevertheless, based on predictions from SwissADME and ADMET-AI, all molecules should be considered to have good gastrointestinal absorption, as clearly illustrated in Figure 3, where the BOILED-Egg model shows the probability of good gastrointestinal absorption in the white region [43].

Another critical parameter considered was lipophilicity, a well-known factor affecting ADME properties and influencing toxicity-related properties. Table 4 summarizes both chromatographically determined and calculated properties. Generally, based on the calculated logP or logD, these molecules exhibited low and moderate lipophilicity, which can be considered promising. Nevertheless, the substantial variations in calculated logP values, such as those for molecule **3**, which range from 1.11 (Percepta) to 5.36 (XlogP3), have raised some concerns. Therefore, in the next step of our investigation, we decided to determine lipophilicity using a well-known protocol described by Valko and co-workers and validated at GSK [44,45]. This protocol, grounded in a gradient chromatographic experiment, has been effectively implemented in our laboratory. Experimentally determined lipophilicity, expressed as chrom logD, is significantly higher than the predicted values. This impacted the Property Forecast Index (PFI), the sum of the number of aromatic rings (#Ar) and chrom logD, used to reflect the principle of simultaneous minimization of lipophilicity and aromaticity, which supports the selection of clinical candidates at GSK [46,47]. Due to relatively high lipophilicity, the PFI value for most proposed molecules is near 7, which is the threshold in this assay. Importantly, molecule **5**, which has promising anticancer activity, showed one of the lowest chrom logD and PFI values below the 7 threshold.

Given the high lipophilicity, which raises concerns about a strong attraction to biological membranes and the potential for baseline toxicity due to their destabilization [48,49], the next step involved examining the affinity of target molecules for phospholipids through artificial membrane chromatography. This protocol is well-established with a threshold for phospholipidosis (>50 CH_IIAM_) [50]. Among the investigated library, only two molecules, **3** and **6**, exceeded that threshold.

## 3. Materials and Methods

### 3.1. Chemistry

All primary substrates were purchased commercially from various sources. The solvents used for column chromatography and thin-layer chromatography (TLC) had purity above 99.5% and were purchased from Chempur (Piekary Śląskie, Polska). Cyanuric chloride (AcrosOrganics, Antwerpen, Belgium), amines, PTCs, and organic bases were purchased from Acros (Antwerpen, Belgium), Ambeed (USA), and Merck (Darmstadt, Germany). Inorganic bases were purchased from Avantor. Solvents for LC-MS were purchased from Merck. ^1^H spectra were recorded using Bruker 400 MHz systems with TMS as an internal standard. Melting points were determined with the Boetius apparatus. Analytical thin-layer chromatography (TLC) was performed using 0.2 mm silica gel precoated aluminum sheets (60 F254, Merck), and UV light at 254 nm was used for visualization. A CEM Discover microwave reactor (P = 50 W with max pressure of 3 bar) or ultrasonic reactor (Sonicator VCX 130 PB, Newtown, USA, amplitude = 70%) was used for the synthesis of compounds **3–11**. LC-HRMS analyses were performed on a Shimadzu Nexera LC-40 XS (Kyoto, Japan) system equipped with PDA (SPD-M40) and LCMS-9030 (QTOF) detectors. Analyses were performed on a Shim-pack Scepter C18, 3 µm; 100 × 2.1 mm column with a gradient of solvents as the mobile phase: Solvent A (water) and B (acetonitrile); t = 0 min, 5% of B, t = 6 min, 95% of B, t = 7 min, 95% of B, t = 7.1 min 5% of B, stop time 10 min; flow rate 0.4 mL min^−1^; UV–VIS detection was performed in a range of 190–700 nm, and MS data were collected in the ESI + mode in a TOF *m*/*z* range of 100–1000 with a scan speed of 15,000 u/s and an event time of 0.1 s. UPLC-MS system: Waters Acquity Premier (Waters Corporation, Milford, MA, USA) was coupled to a Waters Xevo TQ-S Cronos mass spectrometer (electrospray ionization mode: ESI). Chromatographic separations were carried out using an Acquity UPLC BEH (bridged ethylene hybrid) C18 column, 2.1 × 100 mm, and 1.7 µm particle size, equipped with an Acquity UPLC BEH C18 VanGuard pre-column, 2.1 × 5 mm, and 1.7 µm particle size. The column was maintained at 40 °C and eluted under gradient conditions using 95% to 0% of eluent A over 10 min; afterwards 100% of eluent B was eluted over 3 min at a flow rate of 0.3 mL min^−1^. Eluent A: water/formic acid (0.1%, *v*/*v*); eluent B: acetonitrile/formic acid (0.1%, *v*/*v*). Chromatograms were recorded using a Waters eλ PDA detector. Spectra were analyzed in the 200–700 nm range with 1.2 nm resolution and a sampling rate of 20 points/s. MS detection settings of the Waters Xevo TQ-S Cronos mass spectrometer (Massachusetts, USA) were as follows: source temperature of 150 °C, desolvation temperature of 350 °C, desolvation gas flow rate of 600 L h^−1^, cone gas flow of 100 L h^−1^, capillary potential of 3.00 kV, and cone potential of 30 V. Nitrogen was used as both nebulizing and drying gas. The data were obtained in a scan mode ranging from 50 to 1000 *m*/*z* in 0.5 s time intervals. Data acquisition software was MassLynx V 4.2 (Waters, MA, USA).

### 3.2. General Procedure for the Synthesis of Compound ***3*** (Reaction Number 1–14) and Final Compounds ***4***–***11*** (Microwave Synthesis)

Intermediate **1** (0.25 g, 0.77 mmol), the base (2.30 mmol), and PTC (0.08 mmol) were ground in a mortar and transferred to sealed tube, which was previously charged with 2-phenylethylamine **2** (1.92 mmol) or appropriate amine: aniline **2a** (1.92 mmol), 1-phenylmethanamine **2b** (1.92 mmol), 3-phenylpropan-1-amine **2c** (1.92 mmol), 2-[4-(4-fluorophenyl)piperazin-1-yl]ethan-1-amine **2d** (1.92 mmol), 1-phenylpiperazine **2e** (1.92 mmol), 3-(piperazin-1-yl)-1,2-benzothiazole **2f** (1.92 mmol), N^1^-phenylethane-1,2-diamine **2g** (1.92 mmol), and 2-phenoxyethan-1-amine **2h** (1.92 mmol). Subsequently, 0.5 mL of an appropriate solvent was added. The mixture was reacted in the CEM’s Discover microwave reactor for 2.5 min. Reaction progress was monitored via TLC (chloroform: MeOH 95:5 *v*/*v*). After this time, crude product was extracted with DCM/H2O system and then purified by column chromatography on silica gel (chloroform:MeOH 99:1–95:5) and analyzed by LC-HRMS.

#### 3.2.1. *N*^2^-(2-chlorophenyl)-6-morpholino-*N*^4^-phenethyl-1,3,5-triazine-2,4-diamine hydrochloride (**3**) Was Isolated as Follows

White solid, 86.9% yield, mp: 196 °C; ^1^H NMR (400 MHz, MeOD) δ 7.71 (d, *J* = 5.4 Hz, 1H)-Ar, 7.58 (d, *J* = 8.1 Hz, 1H)-Ar, 7.46–7.37 (m, 2H)-Ar, 7.34–7.29 (m, 2H)-Ar, 7.24 (dd, *J* = 14.1, 6.9 Hz, 3H)-Ar, 3.85 (br-d, *J* = 37.0 Hz, 4H)-morph (2x -CH_2_-), 3.77–3.71 (m, 6H) superimposed signals: moph (2x -CH_2_-) and alkyl (-CH_2_-), 2.94 (t, *J* = 6.9 Hz, 3H)-CH_2_-). LC analysis: t—6.514 min, 96.2% purity, ^13^C NMR (101 MHz, DMSO) δ 139.35, 139.04, 137.91, 130.25, 130.10, 129.22, 129.10, 129.07, 128.87, 128.23, 127.92, 127.17, 126.75, 44.53, 42.33, 35.04, 34.49, HRMS (ESI) calc. for C_21_H_23_ClN_6_O [M + H] *m*/*z* 411,1694, found *m*/*z* 411.1694, FT-IR: 3421 (N-H, Str), 3156, 3042 (C-H Ar, Str), 2964, 2855, 2751 (C-H Aliph, Str), 1634 (C-H Ar, Bend), 1608, 1586 (C=N, Str), 1519 (N-H, Bend), 1465, 1440, 1392 (-CH_2_, Bend), 1351, 1302, 1283, 1271 (C-N Ar, Str), 1151, 1117 (C-O, Str), 1059, 1021 (C-N, Str), 990, 968, 933, 893 (C=C Ar, Bend), 841, 803, 770, 745, 731, 699, 632 (C-Cl, Str).

#### 3.2.2. *N*^2^-(2-chlorophenyl)-6-morpholino-*N*^4^-phenyl-1,3,5-triazine-2,4-diamine hydrochloride (**4**) Was Isolated as Follows

White solid, 80% yield, mp: 195 °C, ^1^H NMR (400 MHz, MeOD) δ 7.87 (d, *J* = 5.2 Hz, 1H)-Ar, 7.55 (dd, *J* = 18.8, 7.9 Hz, 3H)-Ar, 7.47–7.41 (m, 3H)-Ar, 7.37–7.24 (m, 2H)-Ar, 3.87 (br-s, 4H) -morph (2x -CH_2_-), 3.76 (br-s, 4H)-morph (2x -CH_2_-). ^13^C NMR (101 MHz, DMSO) δ 160.57, 149.48, 138.45, 134.64, 130.09, 129.13, 128.69, 128.43, 128.01, 127.62, 124.06, 121.32, 66.08, 44.60. UHPL-MS analysis: t–6.274 min, 96.2% purity, calc. for C_19_H_19_ClN_6_O *m*/*z* 382.85, found *m*/*z* 383.14 [M + H], FT-IR: 3291 (N-H, Str), 2974, 2908, 2846 (C-H Aliph, Str), 1622 (C-H Ar, Bend), 1580, 1532 (N-H, Bend), 1464, 1443 (-CH_2_, Bend), 1343, 1316, 1299, 1268 (C-N Ar, Str), 1157, 1112 (C-O, Str), 1063, 1050, 1026 (C-N, Str), 901 (C=C Ar, Bend), 842, 811, 771, 756, 734, 711 (C-Cl, Str).

#### 3.2.3. *N*^2^-benzyl-*N*^4^-(2-chlorophenyl)-6-morpholino-1,3,5-triazine-2,4-diamine hydrochloride (**5**) Was Isolated as Follows

White solid, 70% yield, mp: 212 °C, ^1^H NMR (400 MHz, MeOD) δ 7.76 (d, *J* = 7.6 Hz, 1H)-Ar, 7.57 (d, *J* = 7.9 Hz, 1H)-Ar, 7.46–7.30 (m, 7H)-Ar, 4.65 (s, 2H) -CH_2_-, 3.85 (br-d, *J* = 42.7 Hz, 4H)-morf (2x -CH_2_-), 3.70 (br-s, 4H)-morf (2x -CH_2_-), ^13^C NMR (101 MHz, DMSO) δ 155.48, 138.65, 138.37, 134.35, 133.47, 130.24, 130.04, 128.85, 128.16, 127.97, 127.84, 127.68, 66.09, 44.62, 44.24. LC analysis: t—6.247 min, 99.6% purity, HRMS (ESI) calc. for C_20_H_21_ClN_6_O *m*/*z* 397.1538 [M + H], found *m*/*z* 397.1538. FT-IR: 3150, 3055 (N-H, Str), 2960, 2842 (C-H Aliph, Str), 1644 (C-H Ar, Bend), 1613 (C=N, Str), 1584, 1532 (N-H, Bend), 1462, 1455, 1440 (-CH_2_, Bend), 1377, 1345, 1316, 1295, 1282, 1242, 1232 (C-N Ar, Str), 1114 (C-O, Str), 1076, 1063, 1053, 1026 (C-N, Str), 948, 914, 881 (C=C Ar, Bend), 841, 801, 753, 737, 695 (C-Cl, Str).

#### 3.2.4. *N*^2^-(2-chlorophenyl)-6-morpholino-*N*^4^-(3-phenylpropyl)-1,3,5-triazine-2,4-diamine hydrochloride (**6**) Was Isolated as Follows

White solid, 52% yield, mp: 168 °C, ^1^H NMR (400 MHz, MeOD) δ 7.75 (d, *J* = 7.7 Hz, 1H)-Ar, 7.58 (d, *J* = 7.7 Hz, 1H)-Ar, 7.43 (t, *J* = 7.2 Hz, 1H)-Ar, 7.37 (d, *J* = 7.4 Hz, 1H)-Ar, 7.28 (d, *J* = 7.3 Hz, 2H)-Ar, 7.20 (dd, *J* = 15.3, 7.1 Hz, 3H)-Ar, 3.77 (br-s, 4H)-morf (2x -CH_2_-), 3.70 (d, *J* = 4.5 Hz, 4H)-morf (2x -CH_2_-), 3.47 (t, *J* = 7.2 Hz, 2H) -CH_2_-, 2.72 (t, *J* = 7.3 Hz, 2H, -CH_2_-), 2.02–1.93 (m, 2H, -CH_2_-), ^13^C NMR (101 MHz, DMSO) δ 141.88, 138.57, 133.50, 130.26, 130.06, 128.85, 128.79, 128.74, 128.23, 127.85, 126.28, 66.07, 44.51, 32.73, 30.97, 30.57. LC analysis: t—6.717 min, 97.5% purity, HRMS (ESI) calc. for C_22_H_25_ClN_6_O *m*/*z* 425.1851 [M + H], found *m*/*z* 425.1852. FT-IR: 3420, 3357, 3199, 3160, 3125, 3013 (N-H, Str), 2975, 2945, 2887, 2847, 2759 (C-H Aliph, Str), 1646 (C-H Ar, Bend), 1612 (C=N, Str), 1590, 1533 (N-H, Bend), 1485, 1456, 1441 (-CH_2_, Bend), 1379, 1341, 1298, 1282, 1264, 1237 (C-N Ar, Str), 1116 (C-O, Str), 1087, 1060, 1037, 1022 (C-N, Str), 970, 943, 896 (C=C Ar, Bend), 850, 795, 744, 752, 698 (C-Cl, Str).

#### 3.2.5. *N*^2^-(2-chlorophenyl)-*N*^4^-(2-(4-(4-fluorophenyl)piperazin-1-yl)ethyl)-6-morpholino-1,3,5-triazine-2,4-diamine hydrochloride (**7**) Was Isolated as Follows

White solid, 81.5% yield, mp: 175 °C, ^1^H NMR (600 MHz, DMSO-*d*_6_) δ 11.51 (s, 1H), 10.30 (s, 1H), 8.92 (s, 1H), 7.79 (d, *J* = 8.0 Hz, 1H), 7.57 (d, *J* = 8.0 Hz, 1H), 7.41 (t, *J* = 7.8 Hz, 1H), 7.30 (t, *J* = 7.8 Hz, 1H), 7.11 (t, *J* = 8.8 Hz, 2H), 7.04 (dd, *J* = 9.2, 4.6 Hz, 2H), 3.93–3.83 (m, 3H), 3.70 (d, *J* = 52.9 Hz, 10H), 3.35 (s, 2H), 3.21 (d, *J* = 9.0 Hz, 4H). ^13^C NMR (151 MHz, DMSO) δ 157.90, 156.33, 155.21, 146.79, 133.36, 130.27, 130.15, 128.22, 127.62, 118.39, 118.34, 116.07, 115.93, 65.93, 54.26, 51.23, 46.46, 44.80, 35.46. UHPLC-MS analysis: t—4.055 min, 99.2% purity, calc. for C_25_H_30_ClFN_8_O *m*/*z* 513.02, found *m*/*z* 513.23 [M + H]. FT-IR: 3351, 3216, 3129, 3060 (N-H, Str), 2981, 2863 (C-H Aliph, Str), 1646 (C-H Ar, Bend), 1620 (C=N, Str), 1586, 1536, 1508 (N-H, Bend), 1462, 1438 (-CH_2_, Bend), 1353, 1298, 1280, 1227 (C-N Ar, Str), 1173 (C-F, Str), 1116 (C-O, Str), 1072, 1025 (C-N, Str), 970, 940 (C=C Ar, Bend), 895, 888, 841, 810, 748 (C-Cl, Str).

#### 3.2.6. *N*-(2-chlorophenyl)-4-morpholino-6-(4-phenylpiperazin-1-yl)-1,3,5-triazin-2-amine hydrochloride (**8**) Was Isolated as Follows

White solid, 95.4% yield, mp: 199 °C, ^1^H NMR (600 MHz, DMSO-*d*_6_) δ 9.78 (s, 1H), 7.87 (d, *J* = 8.0 Hz, 1H), 7.54 (d, *J* = 7.9 Hz, 1H), 7.41–7.31 (m, 5H), 7.23 (t, *J* = 7.6 Hz, 1H), 7.08 (s, 1H), 4.06 (s, 4H), 3.77 (s, 4H), 3.66 (t, *J* = 4.8 Hz, 4H), 3.39 (t, *J* = 5.1 Hz, 4H); ^13^C NMR (151 MHz, DMSO) δ 158.01, 147.49, 133.83, 129.60, 129.40, 127.48, 126.62, 126.54, 126.09, 122.92, 118.03, 65.65, 50.05, 44.11, 42.33. LC analysis: t—7.175 min, 100.0% purity, HRMS (ESI) calc. for C_23_H_26_ClN_7_O *m*/*z* 452.1960 [M + H], found *m*/*z* 452.1960. FT-IR: 3458, 3212, 3018 (N-H, Str), 2861, 2654 (C-H Aliph, Str), 1651 (C-H Ar, Bend), 1607 (C=N, Str), 1574, 1539 (N-H, Bend), 1488, 1460, 1442 (-CH_2_, Bend), 1386, 1346, 1303, 1267, 1247 (C-N Ar, Str), 1115 (C-O, Str), 1063, 1021, 1001 (C-N, Str), 920, 877 (C=C Ar, Bend), 853, 842, 792, 754, 695 (C-Cl, Str).

#### 3.2.7. 4-(4-(benzo[d]isothiazol-3-yl)piperazin-1-yl)-*N*-(2-chlorophenyl)-6-morpholino-1,3,5-triazin-2-amine hydrochloride (**9**) Was Isolated as Follows

White solid, 59% yield, mp: 187 °C, ^1^H NMR (600 MHz, DMSO-*d*_6_) δ 9.97 (s, 1H), 8.16 (d, *J* = 8.2 Hz, 1H), 8.08 (d, *J* = 8.1 Hz, 1H), 7.90 (d, *J* = 8.1 Hz, 1H), 7.61–7.54 (m, 2H), 7.46 (t, *J* = 7.6 Hz, 1H), 7.41 (t, *J* = 7.7 Hz, 1H), 7.25 (t, *J* = 7.7 Hz, 1H), 4.01 (s, 4H), 3.79 (s, 4H), 3.68 (t, *J* = 4.8 Hz, 4H), 3.56 (s, 4H), ^13^C NMR (151 MHz, DMSO) δ 163.42, 157.31, 152.54, 134.01, 130.13, 128.50, 128.01, 127.67, 127.17, 127.02, 126.51, 124.99, 124.66, 121.59, 66.07, 49.46, 44.35, 43.88. LC analysis: t—7.517 min, 100.0 % purity, HRMS (ESI) calc. for C_24_H_2_ClFN_8_OS *m*/*z* 509.1633 [M + H], found *m*/*z* 509.1635. FT-IR: 3389, 3211 (N-H, Str), 2968, 2917, 2844 (C-H Aliph, Str), 1649 (C-H Ar, Bend), 1604 (C=N, Str), 1574, 1540 (N-H, Bend), 1486, 1462, 1434 (-CH_2_, Bend), 1379, 1342, 1302, 1256 (C-N Ar, Str), 1113 (C-O, Str), 1053, 1038, 1016, 1001 (C-N, Str), 925, 897 (C=C Ar, Bend), 857, 805, 762, 735 (C-Cl, Str).

#### 3.2.8. *N*^2^-(2-chlorophenyl)-6-morpholino-*N*^4^-(2-(phenylamino)ethyl)-1,3,5-triazine-2,4-diamine hydrochloride (**10**) Was Isolated as Follows

White solid, 76.7% yield, mp: 201 °C, ^1^H NMR (600 MHz, DMSO-*d*_6_) δ 10.26 (s, 1H), 8.65 (d, *J* = 107.3 Hz, 2H), 7.76 (d, *J* = 8.0 Hz, 1H), 7.58 (d, *J* = 8.0 Hz, 1H), 7.41 (t, *J* = 7.8 Hz, 1H), 7.36 (t, *J* = 7.4 Hz, 2H), 7.32–7.24 (m, 2H), 7.16–6.93 (m, 2H), 3.75–3.59 (m, 10H), 3.38 (t, *J* = 6.6 Hz, 2H), ^13^C NMR (151 MHz, DMSO) δ 155.30, 134.22, 133.38, 130.27, 130.09, 129.94, 129.85, 128.23, 128.01, 127.76, 127.58, 119.01, 66.11, 46.21, 44.55, 38.02. LC analysis: t—6.125 min, 99.5% purity, HRMS (ESI) calc. for C_21_H_24_ClN_7_O *m*/*z* 426.1804 [M + H], found *m*/*z* 426.1852. FT-IR: 3198, 3162, 3124, 3080 (N-H, Str), 2956, 2913, 2679, 2614 (C-H Aliph, Str), 1645 (C-H Ar, Bend), 1611 (C=N, Str), 1587, 1529 (N-H, Bend), 1495, 1463, 1443 (-CH_2_, Bend), 1377, 1362, 1341, 1302, 1277 (C-N Ar, Str), 1113 (C-O, Str), 1059, 1036, 1018 (C-N, Str), 963, 938, 899 (C=C Ar, Bend), 856, 810, 776, 746, 689 (C-Cl, Str).

#### 3.2.9. *N*^2^-(2-chlorophenyl)-6-morpholino-*N*^4^-(2-phenoxyethyl)-1,3,5-triazine-2,4-diamine hydrochloride (**11**) Was Isolated as Follows

White solid, 54% yield, mp: 136 °C; ^1^H NMR (400 MHz, DMSO-d6) δ 9.89 (d, *J* = 139.5 Hz, 1H), 8.54 (s, 1H), 7.75 (d, *J* = 8.1 Hz, 1H), 7.55 (dd, *J* = 17.0, 8.0 Hz, 1H), 7.35 (dt, *J* = 43.2, 7.7 Hz, 4H), 6.95 (t, *J* = 7.0 Hz, 3H), 4.11 (dt, *J* = 18.7, 5.6 Hz, 4H), 3.81–3.63 (m, 8H), ^13^C NMR (101 MHz, DMSO) δ 158.63, 156.26, 151.38, 137.16, 134.72, 133.66, 130.26, 130.00, 128.25, 127.84, 127.31, 121.28, 115.00, 104.15, 66.14, 65.95, 44.48. LC analysis: t—6.439 min, 99% purity, HRMS (ESI) calc. for C_21_H_23_ClN_6_O_2_ m/z 427.1644 [M + H], found m/z 427.1642. FT-IR: 3409, 3197, 3009 (N-H, Str), 2960, 2937, 2855 (C-H Aliph, Str), 1640 (C-H Ar, Bend), 1613 (C=N, Str), 1585, 1540 (N-H, Bend), 1497, 1458, 1440 (-CH_2_, Bend), 1387, 1364, 1341, 1300, 1285 (C-N Ar, Str), 1250, 1171, 1109 (C-O, Str), 10,582, 1063, 1029 (C-N, Str), 924, 891, 874 (C=C Ar, Bend), 847, 811, 794, 749, 68s9 (C-Cl, Str).

#### 3.2.10. Procedure for the Synthesis of Compound **13**

Cyanuric chloride (**12**) (5.00 g; 0.03 mol) was weighed into a 150 mL round-bottom flask and dissolved in 52 mL of tetrahydrofuran (THF). Then, 2.9 mL of 2-chloroaniline (0.03 mol) (**13**) and 5.2 mL (0.03 mol) of *N,N*-diisopropylethylamine were weighed into two separate beakers, respectively. The whole concentration was cooled in an ice bath to 0 °C. Then, the remaining reagents were added dropwise, constantly monitoring that the temperature did not exceed 5 °C. The mixture was left for 2 h. After the reaction was complete (TLC control), the mixture was transferred to a crystallizer and left to dry. The dry crude product was dissolved in 60 mL of dichloromethane, transferred to a separatory funnel, and washed with 40 mL of water with the addition of 1 M hydrochloric acid. The organic phase was separated from the aqueous phase, dried over magnesium sulfate, and left to dry. The precipitate was triturated in cold methanol and then filtered to give pure product, which did not require further purification.

4,6-Dichloro-N-(2-chlorophenyl)-1,3,5-triazin-2-amine (**13**) Was Isolated as Follows:

White solid, 49% yield, mp: 162–167 °C, ^1^H NMR (400 MHz, DMSO) δ 11.02 (s, 1H)-NH-, 7.58 (dd, *J* = 7.8, 1.5 Hz, 1H)-Ar, 7.52 (dd, *J* = 7.8, 1.6 Hz, 1H)-Ar, 7.42 (td, *J* = 7.6, 1.6 Hz, 1H)-Ar, 7.36 (td, *J* = 7.6, 1.7 Hz, 1H)-Ar.

#### 3.2.11. Procedure for the Synthesis of Compound **1**

4,6-dichloro-*N*-(2-chlorophenyl)-1,3,5-triazin-2-amine (**13**) (3.6 g; 0.01 mol) was weighed into a 250 mL round-bottom flask and dissolved in 125 mL of tetrahydrofuran (THF). Then, 1.9 mL (0.02 mol) of morpholine (**14**) and 3.5 mL (0.01 mol) of *N,N*-diisopropylethylamine (DIPEA) were measured into two separate beakers, respectively. The round-bottom flask was cooled in an ice bath, and, after reaching 0 °C, the remaining reagents were added dropwise, constantly monitoring that the temperature did not exceed 0 °C. After all the substrates had been added dropwise, the solution was stirred at room temperature for 24 h. After the reaction was complete (TLC control), the mixture was transferred to a crystallizer and left to dry. The crude product was dissolved in 100 mL of dichloromethane and washed with 50 mL of water with the addition of 1M hydrochloric acid solution. The organic phase was separated from the aqueous phase, dried over magnesium sulfate, and left to dry. The crude product was purified by column chromatography using the eluent chloroform—methanol in volume ratios of 99:1–95:5 to obtain pure product.

4-Chloro-*N*-(2-chlorophenyl)-6-(morpholin-4-yl)-1,3,5-triazin-2-amine (**1**) Was Isolated as Follows:

White solid, 37.8% yield, mp: 171–176 °C, ^1^H NMR (400 MHz, DMSO) δ 9.65 (s, 1H)-NH-, 7.57–7.50 (m, 2H)-Ar, 7.36 (td, *J* = 7.8, 1.3 Hz, 1H)-Ar, 7.26 (td, *J* = 7.8, 1.4 Hz, 1H)-Ar, 3.69–3.58 (m, 8H)-morf (4x -CH_2_-). UHPLC-MS analysis: t—8.03 min, 98.6% purity, calc. for C_13_H_13_Cl_2_N_5_O *m*/*z* 326,18, found *m*/*z* 325,34 [M − H].

#### 3.2.12. General Procedure for the Synthesis of Final Compounds **3**, **5**, **6**, **8**–**11** (Sonochemistry)

Intermediate **1** (0.25 g, 0.77 mmol), the base (2.30 mmol), and PTC (0.08 mmol) were ground in a mortar and transferred to sealed tube, which was previously charged with 2.5 eq of appropriate amine **2b**–**2g**. Subsequently, 0.5 mL of water and 5% of wt DMF were added. The mixture was reacted in the ultrasonic reactor (Sonicator VCX 130 PB) for 2.5 min. Reaction progress was monitored via TLC (chloroform/MeOH 95:5 *v*/*v*). After this time, crude product was extracted with DCM/H2O system and then purified by column chromatography on silica gel (chloroform/MeOH 99:1–95:5) and analyzed by LC-HRMS.

### 3.3. In Vitro Screening and ADME Evaluation

The human colorectal cancer cell lines SW620, SW480 were obtained from the American Type Culture Collection (ATCC). Both cell lines were cultured in Dulbecco’s Modified Eagle Medium (DMEM) (Capricorn Scientific GmbH, Manassas, USA), which was supplemented with 10% fetal bovine serum (FBS) and 50 µg/mL of gentamicin. Cells were incubated at 37 °C in a humidified atmosphere containing 5% CO_₂_. The cultures were passaged approximately every three days upon reaching confluence using a 0.25% trypsin-EDTA solution, at a ratio of approximately 1:3 to 1:4. For the MTS assay, cells were seeded at a density of 10,000 cells per well in 96-well plates. After 24 h, compounds were added at five concentrations (100, 50, 25, 12.5, and 6.125 µM), and cells were incubated for an additional 24 h. Cytotoxicity was assessed using the CellTiter 96^®^ AQueous One Solution Cell Proliferation Assay (Promega), following the manufacturer’s instructions. Absorbance was measured at 490 nm using a microplate reader.

The IC₅₀ values were determined relative to the untreated control cells (without test compounds). All experiments were performed in triplicate (n = 3). Compounds for which IC₅₀ values could not be reliably determined were excluded from quantitative analysis. This included cases where the tested compounds showed no significant inhibitory activity within the tested concentration range or where the variability between replicates precluded accurate curve fitting. Such compounds are reported as ‘>100µM’ or ‘>100µM*’, respectively, in the results.

#### 3.3.1. In Silico Tools

Using ADMET-AI (accessed on 14 April 2025), SwissADME (accessed on 14 April 2025), and ACDlabs Perceta module, a set of theoretical ADMET properties was calculated and collected in Table S1.

#### 3.3.2. Lipophilicity and Phospholipids Affinity Assays

All chromatographic experiments adhered to the protocols established by Valko and colleagues [44,45], which were implemented in our laboratory [51,52]. Essentially, biochromatography facilitated the assessment of lipophilicity and phospholipid affinity, represented as Chromatographic Hydrophobicity Index (CHI) values, through a gradient elution experiment, comparing results with model substances. The Prominence-1 LC-2030C 3D HPLC system, operated via LabSolution software (v6.81, Kyoto, Japan), was utilized for all chromatographic analyses. Throughout the research, distinct chromatographic columns were employed: IAM.PC.DD_2_ (10 × 4.6 mm × 10.0 µm with a guard column; Regis Technologies; USA) and C_18_ Hypersil GOLD^TM^ (100 mm × 4.6 mm; 5.0 µm with a guard column; Thermo Scientific, Waltham, MA, USA). Each column was paired with guard columns featuring the same stationary phases as the main column. Mobile phase A consisted of a water solution of 50 mM ammonium acetate (VWR International, Leuven, Belgium), adjusted to physiological pH 7.4 using concentrated ammonia solution (Avantor Performance Materials Poland S.A., Gliwice, Poland). The mobile phase was prepared with ultrapure water from a Milli-Q water purification system (Merck Millipore, Darmstadt, Germany), achieving a resistivity of 18.2 MΩ. For phospholipid binding assessments, mobile phase B was HPLC grade acetonitrile (Chempur, Piekary Śląskie, Poland), with a linear gradient from 0 to 85% B over 5.25 min, then maintained at 85% ACN for 0.5 min. The mobile phase was delivered at a flow rate of 1.5 mL/min, with the IAM.PC.DD2 column temperature held steady at 30 °C. For lipophilicity evaluations, the solvents and flow rate mirrored those used in IAM chromatography. The C18 Hypersil GOLD^TM^ column was kept at 40 °C. Similarly, a linear gradient was applied from 0 to 5.25 min, but, in this instance, it ranged from 2 to 98% ACN, with the maximum phase B concentration sustained for 2.5 min. Dimethyl sulfoxide (Avantor Performance Materials Poland S.A., Poland) was employed to dissolve the target solutes, forming a solution with a concentration of 200 µg/mL. Detection occurred in the UV region at a wavelength of 190–300 nm. The injection volume was 5 μL, and each compound was analyzed in duplicate, with retention time variation below 0.5%. Retention times for the studied molecules and reference standards are detailed in the Supplementary Material, Tables S1 and S2.

Experimentally determined CHI values were converted to chrom log*D* using the following equation [53]:

Chrom log*D* = 0.0525 × CHI − 1.467 (1)

In addition, the Property Forecast Index (PFI), the sum of the number of aromatic rings(#Ar), and a lipophilicity measure are used to reflect the principle of simultaneous minimization of lipophilicity and aromaticity [46], which supports the selection of oral clinical candidates in GKS [47].

PFI = Chrom log*D*_7.4_ + #Ar (2)

## 4. Conclusions

This study successfully developed an efficient method for synthesizing 1,3,5-triazine derivatives modified with morpholine using microwave-assisted reactions as well as a sonochemistry protocol. The optimized approach significantly improved reaction efficiency by reducing reaction time, minimizing energy consumption, and achieving high product yields, making it a viable alternative to conventional synthetic methods.

The most effective reaction conditions were identified as the use of sodium carbonate as the base, TBAB as the phase-transfer catalyst, and DMF as the solvent, with microwave irradiation at 150 °C for 150 s. Under these conditions, the synthesis of final derivatives was achieved with yields ranging from 54% to 96%. Notably, the phenylpiperazine derivative exhibited the highest yield, further supporting the efficiency of the optimized protocol. While DMF proved to be the most effective solvent under microwave conditions, its known toxicity and environmental impact limit the greenness of this otherwise efficient method. To address this issue, we successfully reduced the amount of DMF by 95%, replacing it with water, and simultaneously switched the energy source from microwave irradiation to ultrasound. As a result, not only were the reaction yields comparable or even superior to those obtained under microwave conditions, but the overall procedure also became significantly more environmentally friendly. Beyond the chemical optimization, this study also explored the biological activity of the synthesized compounds. Among the tested derivatives, compound **11** exhibited the highest activity against the SW620 cell line, outperforming the reference drug 5-fluorouracil. Additionally, compound **5** demonstrated promising cytotoxic potential, suggesting a favorable influence of benzylamine substitution. These findings provide a strong foundation for further exploration of 1,3,5-triazine derivatives in drug development.

Overall, this work established a sustainable synthetic pathway for 1,3,5-triazine derivatives and identified promising candidates for further biological evaluation. The findings pave the way for future research into refining these methods and exploring the therapeutic potential of these compounds, contributing to the broader pursuit of eco-friendly practices in medicinal chemistry.

## Figures and Tables

**Table 1:** Evaluation of the effect based on obtaining final product **3.** MW, 2.5 min, P = 50 W, and P_max_ = 3 bar.

Reaction No.	Base	PTC	Solvent	Yield [%]
1	Na_2_CO_3_	TBAB	DMF	87
2	K_2_CO_3_			64
3	KOH			48
4	NaOH			23
5	DIPEA			23
6	TEA			56
7	NH_3_∙H_2_O			2
8	Na_2_CO_3_	TEBA	DMF	56
9		TEAC		80
10		CTBA		85
11		KI		44
12		-		53
13		TBAB	H_2_O	10
14			-	8

**Table 2:** Assessment of obtained yields for final compounds under microwave synthesis as well as sonochemistry.

Compound No.	Amines		Yield [%]	
			Microwave Synthesis	Sonochemistry
**3**	**2**	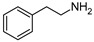	87	90
**4**	**2a**	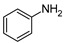	80	-
**5**	**2b**	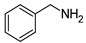	70	89
**6**	**2c**	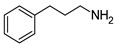	52	85
**7**	**2d**	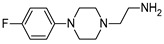	82	-
**8**	**2e**	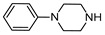	96	87
**9**	**2f**	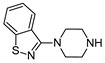	59	94
**10**	**2g**	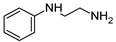	77	41
**11**	**2h**	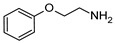	54	69

**Table 3:** Cytotoxicity expressed in IC_50_ [μM] ± SD for compounds **3–11** and reference 5-fluorouracil. For compounds that showed no detectable effect at the highest tested concentration, IC₅₀ is reported as >100 µM.

Compound No.	SW480	SW620
**3**	>100	>100
**4**	>100	36.09 ± 46.48
**5**	43.4 ± 12.22	32.83 ± 23.37
**6**	>100	>100
**7**	>100	>100 *
**8**	20.57 ± 24.87	>100 *
**9**	>100	22.1 ± 17.29
**10**	>100	>100 *
**11**	>100 *	5.85 ± 14.34
5-fluorouracil	15.45 ± 7.825	21.74 ± 2.754

* indicates that IC₅₀ could not be determined due to high variability or lack of a consistent dose–response.

**Table 4:** Chromatographically and computationally determines lipophilicity of investigated 1,3,5-triazine derivatives together with phospholipid affinity and PFI.

Compound No.	RP-HPLC			IAM-HPLC	Perpecpta		SwissADME						ADMET-AI
	chrom logD	Ar	PFI	CHI IAM	clogD	logP	iLOGP	XLOGP3	WLOGP	MLOGP	Silicos-IT Log P	Consensus Log P	logP
**3**	3.95	4	7.95	55.97	2.84	1.11	4.29	5.36	2.90	2.33	3.12	3.60	4.04
**4**	4.10	3	7.10	46.34	3.61	1.80	4.11	4.54	3.02	1.95	2.63	3.25	3.60
**5**	3.95	3	6.95	46.45	3.27	1.26	3.89	4.56	2.87	1.95	2.31	3.12	3.63
**6**	4.71	3	7.71	51.63	2.74	0.81	4.08	4.61	2.29	2.37	2.08	3.09	3.43
**7**	4.44	3	7.44	49.59	4.06	2.51	4.28	5.19	3.58	2.68	3.48	3.84	4.15
**8**	4.15	3	7.15	47.79	3.45	1.63	3.73	3.04	3.47	2.29	2.34	2.97	3.85
**9**	4.23	3	7.23	48.02	3.70	2.07	4.13	4.83	3.19	2.47	3.10	3.54	3.76
**10**	3.94	3	6.94	44.55	2.97	1.86	4.75	4.61	2.57	2.38	2.48	3.36	3.48
**11**	4.01	3	7.01	45.92	3.51	1.66	4.01	4.37	2.99	2.25	2.71	3.27	3.72

## Data Availability

Data is contained within the article or Supplementary Material.

## Supplementary Materials

The following supporting information can be downloaded at https://www.mdpi.com/article/10.3390/molecules30112437/s1: Table S1. Theoretical pharmacokinetic descriptors calculated using ADMETlab AI and SwissADME. Table S2. Retention times of the investigated compounds. Table S3. Retention times of the calibration mixtures.
